# Supplementary material for: A cross-cultural investigation of the short version of the Celebrity Attitude Scale (CAS-7) across five countries
Source: PLoS One. 2025 Sep 11;20(9):e0331696. doi: 10.1371/journal.pone.0331696 (PMC12425179; doi:10.1371/journal.pone.0331696)
Supplement: S1 Table — Note. ES = Entertainment-Social; IP = Intense-Personal; BP = Borderline-Pathological. (DOCX) [file pone.0331696.s001.docx]

**SM Table 1**

Items of the 7-item version of the CAS (CAS-7)

| **Item number** | **Item content** |
| --- | --- |
| 1 (IP2) | I share with my favorite celebrity a special bond that cannot be described in words. |
| 2 (ES1) | It is enjoyable just to be with others who like my favorite celebrity. |
| 3 (BP2) | I often feel compelled to learn the personal habits of my favorite celebrity. |
| 4 (ES2) | I like watching and hearing about my favorite celebrity when I am in a large group of people. |
| 5 (IP1) | When something good happens to my favorite celebrity I feel like it happened to me. |
| 6 (ES3) | Keeping up with news about my favorite celebrity is an entertaining pastime. |
| 7 (BP1) | If someone gave me several thousand dollars to do with as I please, I would consider spending it on a personal possession (like a napkin or paper plate) once used by my favorite celebrity. |

Note. *ES=Entertainment-Social; IP=Intense-Personal; BP=Borderline-Pathological.*
