# Supplementary material for: A cross-cultural investigation of the short version of the Celebrity Attitude Scale (CAS-7) across five countries
Source: PLoS One. 2025 Sep 11;20(9):e0331696. doi: 10.1371/journal.pone.0331696 (PMC12425179; doi:10.1371/journal.pone.0331696)
Supplement: S2 Table — Note. ES = Entertainment-Social; IP = Intense-Personal; BP = Borderline-Pathological. (DOCX) [file pone.0331696.s002.docx]

**SM Table 2**

1-factor model: Factor loadings

| Items | Sample 1: Canadian student n=252 | Sample 2: Hungarian student n=295 | Sample 3: Hungarian fans n=1361 | Sample 4: Indonesian student n=321 | Sample 5: Iranian general n=627 | Sample 6: US student n=570 | Sample 7: US general n=924 |
| --- | --- | --- | --- | --- | --- | --- | --- |
| ES1 | 0.674 | 0.598 | 0.623 | 0.505 | 0.751 | 0.691 | 0.674 |
| ES2 | 0.704 | 0.616 | 0.646 | 0.728 | 0.900 | 0.754 | 0.682 |
| ES3 | 0.635 | 0.575 | 0.647 | 0.709 | 0.808 | 0.727 | 0.668 |
| IP1 | 0.694 | 0.623 | 0.761 | 0.451 | 0.624 | 0.616 | 0.794 |
| IP2 | 0.551 | 0.611 | 0.720 | 0.520 | 0.533 | 0.554 | 0.776 |
| BP1 | 0.401 | 0.509 | 0.561 | 0.321 | 0.406 | 0.407 | 0.715 |
| BP2 | 0.663 | 0.654 | 0.643 | 0.515 | 0.427 | 0.650 | 0.780 |

Note. *ES=Entertainment-Social; IP=Intense-Personal; BP=Borderline-Pathological.*
