# Supplementary material for: A cross-cultural investigation of the short version of the Celebrity Attitude Scale (CAS-7) across five countries
Source: PLoS One. 2025 Sep 11;20(9):e0331696. doi: 10.1371/journal.pone.0331696 (PMC12425179; doi:10.1371/journal.pone.0331696)
Supplement: S3 Table — Note. ES = Entertainment-Social; IP = Intense-Personal; BP = Borderline-Pathological; IPBP = Intense-Pathological. (DOCX) [file pone.0331696.s003.docx]

**SM Table 3**

2-factor model: Factor loadings

| Items | Sample 1: Canadian student n=252 | Sample 2: Hungarian student n=295 | Sample 3: Hungarian fans n=1361 | Sample 4: Indonesian student n=321 | Sample 5: Iranian general n=627 | Sample 6: US student n=570 | Sample 7: US general n=927 |
| --- | --- | --- | --- | --- | --- | --- | --- |
| *ES* | | | | | | | |
| ES1 | 0.777 | 0.671 | 0.696 | 0.535 | 0.808 | 0.722 | 0.776 |
| ES2 | 0.788 | 0.685 | 0.700 | 0.764 | 0.843 | 0.813 | 0.809 |
| ES3 | 0.610 | 0.646 | 0.705 | 0.733 | 0.710 | 0.738 | 0.752 |
| *IPBP* | | | | | | | |
| IP1 | 0.736 | 0.662 | 0.791 | 0.541 | 0.693 | 0.696 | 0.822 |
| IP2 | 0.616 | 0.634 | 0.745 | 0.645 | 0.664 | 0.634 | 0.812 |
| BP1 | 0.431 | 0.557 | 0.573 | 0.360 | 0.500 | 0.483 | 0.751 |
| BP2 | 0.712 | 0.685 | 0.657 | 0.590 | 0.526 | 0.690 | 0.770 |
| Factor correlations | | | | | | | |
| ES-IPBP | 0.740 | 0.744 | 0.815 | 0.702 | 0.830 | 0.760 | 0.754 |

*Note. ES=Entertainment-Social; IP=Intense-Personal; BP=Borderline-Pathological; IPBP=Intense-Pathological.*
