# Supplementary material for: A cross-cultural investigation of the short version of the Celebrity Attitude Scale (CAS-7) across five countries
Source: PLoS One. 2025 Sep 11;20(9):e0331696. doi: 10.1371/journal.pone.0331696 (PMC12425179; doi:10.1371/journal.pone.0331696)
Supplement: S4 Table — Note. rES-IP = correlation between Entertainment-Social and Intense-Personal factors. rES-BP = correlation between Entertainment-Social and Borderline-Pathological factors. rIP-BP = correlation between Intense-Personal and Borderline-Pathological factors. (DOCX) [file pone.0331696.s004.docx]

**SM Table 4**

3-factor model: Factor loadings

| Items | Sample 1: Canadian student n=252 | Sample 2: Hungarian student n=295 | Sample 3: Hungarian fans n=1361 | Sample 4: Indonesian student n=321 | Sample 5: Iranian general n=627 | Sample 6: US student n=570 | Sample 7: US general n=927 |
| --- | --- | --- | --- | --- | --- | --- | --- |
| Entertainment-Social*(ES)* | | | | | | | |
| ES1 | 0.778 | 0.673 | 0.696 | 0.533 | 0.732 | 0.721 | 0.776 |
| ES2 | 0.788 | 0.681 | 0.700 | 0.767 | 0.939 | 0.813 | 0.808 |
| ES3 | 0.610 | 0.647 | 0.705 | 0.731 | 0.814 | 0.740 | 0.754 |
| Intense-Personal *(IP)* | | | | | | | |
| IP1 | 0.746 | 0.695 | 0.805 | 0.569 | 0.652 | 0.723 | 0.840 |
| IP2 | 0.621 | 0.668 | 0.754 | 0.697 | 0.638 | 0.652 | 0.826 |
| Borderline-Pathological*(BP)* | | | | | | | |
| BP1 | 0.432 | 0.590 | 0.615 | 0.382 | 0.769 | 0.478 | 0.762 |
| BP2 | 0.716 | 0.768 | 0.714 | 0.639 | 0.788 | 0.705 | 0.799 |
| Factor correlations | | | | | | | |
| r_ES-IP_ | 0.725 | 0.722 | 0.801 | 0.626 | 0.827 | 0.714 | 0.727 |
| r_ES-BP_ | 0.747 | 0.667 | 0.756 | 0.697 | 0.439 | 0.782 | 0.758 |
| r_IP-BP_ | 0.976 | 0.801 | 0.878 | 0.810 | 0.757 | 0.928 | 0.932 |

Note. r_ES-IP_ = correlation between Entertainment-Social and Intense-Personal factors. r_ES-BP_= correlation between Entertainment-Social and Borderline-Pathological factors. r_IP-BP_= correlation between Intense-Personal and Borderline-Pathological factors.
