# Supplementary material for: A cross-cultural investigation of the short version of the Celebrity Attitude Scale (CAS-7) across five countries
Source: PLoS One. 2025 Sep 11;20(9):e0331696. doi: 10.1371/journal.pone.0331696 (PMC12425179; doi:10.1371/journal.pone.0331696)
Supplement: S5 Table — Note. ES = Entertainment-Social; IP = Intense-Personal; BP = Borderline-Pathological. (DOCX) [file pone.0331696.s005.docx]

**SM Table 5**

Bifactoral model: Factor loadings

| Items | Sample 1: Canadian student n=252 | Sample 2: Hungarian student n=295 | Sample 3: Hungarian fans n=1361 | Sample 4: Indonesian student n=321 | Sample 5: Iranian general n=627 | Sample 6: US student n=570 | Sample 7: US general n=927 |
| --- | --- | --- | --- | --- | --- | --- | --- |
| *ES – Entertainment-Social* | | | | | | | |
| ES1 | 0.836 | 0.420 | 0.499 | 0.492 | 0.202 | 0.211 | 0.122 |
| ES2 | 0.367 | 0.409 | 0.368 | 0.510 | 0.585 | 0.713 | 0.665 |
| ES3 | 0.158 | 0.423 | 0.377 | 0.463 | 0.453 | 0.121 | 0.071 |
| *IPBP – Intense-Pathological* | | | | | | | |
| IP1 | 0.123 | 0.071 | 0.126 | 0.876 | 0.047 | 0.396 | 0.505 |
| IP2 | 0.686 | 0.056 | 0.117 | 0.122 | 0.316 | 0.449 | 0.559 |
| BP1 | 0.159 | 0.897 | -0.225 | -0.085 | 0.696 | 0.414 | 0.556 |
| BP2 | 0.153 | 0.194 | -0.220 | 0.016 | 0.626 | 0.234 | 0.343 |
| *CAS – general factor* | | | | | | | |
| ES1 | 0.548 | 0.532 | 0.542 | 0.310 | 0.729 | 0.651 | 0.744 |
| ES2 | 0.610 | 0.531 | 0.578 | 0.568 | 0.774 | 0.701 | 0.747 |
| ES3 | 0.617 | 0.501 | 0.579 | 0.551 | 0.655 | 0.735 | 0.756 |
| IP1 | 0.733 | 0.661 | 0.796 | 0.482 | 0.695 | 0.574 | 0.644 |
| IP2 | 0.515 | 0.647 | 0.744 | 0.601 | 0.576 | 0.495 | 0.606 |
| BP1 | 0.395 | 0.441 | 0.582 | 0.407 | 0.395 | 0.339 | 0.535 |
| BP2 | 0.673 | 0.633 | 0.669 | 0.600 | 0.431 | 0.637 | 0.695 |

*Note. ES=Entertainment-Social; IP=Intense-Personal; BP=Borderline-Pathological.*
