# Supplementary material for: A cross-cultural investigation of the short version of the Celebrity Attitude Scale (CAS-7) across five countries
Source: PLoS One. 2025 Sep 11;20(9):e0331696. doi: 10.1371/journal.pone.0331696 (PMC12425179; doi:10.1371/journal.pone.0331696)
Supplement: S6 Table — Note. ES=Entertainment-Social;IPBP=Intense-Pathological; CAS7=general factor; ECV=Explained Common Variance: is the proportion of all common variance explained by that factor; Ω=Omega: is model-based estimate of internal reliability of the multidimensional composite; Ωh=Omega Hierarchical: reflects the percentage of systematic variance in unit-weighted total scores that can be attributed to the individual differences ont he general factors; H=represents the correlation between a factor and an optimally-weighted item composite. (DOCX) [file pone.0331696.s006.docx]

**SM Table 6**

Reliability indices of the bifactor model in seven samples.

| Reliability Indices | ECV | | | Ω | | | Ωh | | | H | | |
| --- | --- | --- | --- | --- | --- | --- | --- | --- | --- | --- | --- | --- |
|  | ES | IPBP | CAS7 | ES | IPBP | CAS7 | ES | IPBP | CAS7 | ES | IPBP | CAS7 |
| Sample 1: Canadian student n=252 | 0.22 | 0.14 | 0.64 | 0.82 | 0.76 | 0.86 | 0.30 | 0.19 | 0.73 | 0.71 | 0.49 | 0.81 |
| Sample 2: Hungarian student n=295 | 0.14 | 0.23 | 0.62 | 0.71 | 0.81 | 0.85 | 0.28 | 0.17 | 0.71 | 0.39 | 0.81 | 0.78 |
| Sample 3: Hungarian fans n=1361 | 0.15 | 0.04 | 0.82 | 0.75 | 0.81 | 0.87 | 0.26 | 0.00 | 0.80 | 0.40 | 0.12 | 0.85 |
| Sample 4: Indonesian student n=321 | 0.21 | 0.26 | 0.55 | 0.73 | 0.71 | 0.81 | 0.37 | 0.12 | 0.65 | 0.49 | 0.77 | 0.73 |
| Sample 5: Iranian general n=627 | 0.14 | 0.23 | 0.63 | 0.88 | 0.80 | 0.89 | 0.22 | 0.31 | 0.72 | 0.45 | 0.63 | 0.84 |
| Sample 6: US student n=570 | 0.15 | 0.35 | 0.69 | 0.85 | 0.73 | 0.86 | 0.17 | 0.26 | 0.72 | 0.52 | 0.41 | 0.82 |
| Sample 7: US general n=927 | 0.10 | 0.21 | 0.69 | 0.87 | 0.87 | 0.92 | 0.11 | 0.34 | 0.76 | 0.45 | 0.58 | 0.87 |

*Note. ES=Entertainment-Social; IPBP=Intense-Pathological; CAS7=general factor; ECV=Explained Common Variance: is the proportion of all common variance explained by that factor; Ω=Omega: is model-based estimate of internal reliability of the multidimensional composite; Ωh=Omega Hierarchical: reflects the percentage of systematic variance in unit-weighted total scores that can be attributed to the individual differences on the general factors; H=represents the correlation between a factor and an optimally-weighted item composite.*
