# Supplementary material for: A cross-cultural investigation of the short version of the Celebrity Attitude Scale (CAS-7) across five countries
Source: PLoS One. 2025 Sep 11;20(9):e0331696. doi: 10.1371/journal.pone.0331696 (PMC12425179; doi:10.1371/journal.pone.0331696)
Supplement: S7 Table — Note. Chi-square test: χ²(12, n = 4353) = 1035.04, p < 0.001; Identical letters in the same row indicate nonsignificant (p> 0.05) difference between groups, while different letters indicate significant group differences (p< 0.05) according to the post-hoc z-test. (DOCX) [file pone.0331696.s007.docx]

**SM Table 7**

Chi-square test with post-hoc z-tests for gender differences across study-samples

| gender | Sample 1: Canadian student n=252 | Sample 2: Hungarian student n=295 | Sample 3: Hungarian fans n=1361 | Sample 4: Indonesian student n=321 | Sample 5: Iranian general n=627 | Sample 6: US student n=570 | Sample 7: US general n=927 |
| --- | --- | --- | --- | --- | --- | --- | --- |
| male | 95 (6.2%)a,b | 83 (5.4%)b | 270 (17.6%)c | 48 (3.1%)c | 457 (29.8%)d | 160 (10.4%)b | 421 (27.4%)a |
| female | 154 (5.5%)a,b | 212 (7.6%)b | 1091 (38.9%)c | 273 (9.7%)c | 166 (5.9%)d | 408 (14.5%)b | 501 (17.9%)a |
| other | 3 (21.4%)a | 0 (0%)a,b | 0 (0%)b | 0 (0%)a,b | 4 (28.6%)a,b | 2 (14.3%)b | 5 (35.7%)a,b |

*Note. Chi-square test: χ²(12, n = 4353) = 1035.04, p<0.001; Identical letters in the same row indicate nonsignificant (p> 0.05) difference between groups, while different letters indicate significant group differences (p< 0.05) according to the post-hoc z-test.*
