# Supplementary material for: A cross-cultural investigation of the short version of the Celebrity Attitude Scale (CAS-7) across five countries
Source: PLoS One. 2025 Sep 11;20(9):e0331696. doi: 10.1371/journal.pone.0331696 (PMC12425179; doi:10.1371/journal.pone.0331696)
Supplement: Attachment — Submitted filename: Response_to_Reviewers_CAS7.docx [file pone.0331696.s009.docx]

Dear Osmond Ekwebelem,

We are grateful for the positive and constructive comments. The insightful suggestions helped us significantly improve the quality of our manuscript. Following your recommendation, we are pleased to submit a revised version of the manuscript entitled ***“A cross-cultural investigation of the short version of the Celebrity Attitude Scale (CAS-7) across five countries”*** for further consideration in ***PlosOne***.

We have addressed all the comments (summarized below), which you can see in the revised version of the manuscript with yellow highlights. Reviewer comments appear in **bold**, followed by our response, and the revised section of the manuscript (in *italics*).

Again, we are grateful for all the recommendations. We hope that the revisions will meet your standards for publication in *PlosOne*.

Sincerely,

the authors

**Editor**

**Comments**

**1.Some sentences are unnecessarily long and complex. Breaking them down would improve readability.
Example: "Therefore, based on the findings by Zsila et al. (14), the CAS-7 is fundamentally unidimensional with some multidimensionality." Simplify or rephrase to avoid confusion between "fundamentally unidimensional" and "some multidimensionality."**

Author’s response:

Thank you for bringing this to our attention. We have made the necessary modifications in the manuscript accordingly.

**Changes in themanuscript:**

Page 8:

*“Therefore, based on the findings by Zsila et al. (14), the CAS-7 is mainly unidimensional.”*

Page 20:

*“Therefore, the present findings also indicated that the CAS-7 is primarily a unidimensional construct, consistent with Zsila et al. (14).”*

**2.Some minor errors, like missing commas or awkward phrasing.
Examples:
o Line 8: "Cronbach’s 123 alphas" = Shouldjust be "Cronbach’s alphas."
o Line 9: "which have been generally used in celebrity worship research." It could be smoother as "which are commonly used in celebrity worship research."**

Author’s response:

We carefully revised and rewrite those lines accordingly.

**Changes in themanuscript:**

Page 8:

„*Common problems concerning the CAS were its length, the lack of a cut-off score to differentiate between healthy and problematic celebrity admiration (13), the poorer internal consistency of the BP factor (Cronbach’s alphas were around 0.6 or below across many studies, see [10]), and the high correlation between the IP and BP factors (14), which have been regarded as the two problematic factors, while ES theoretically represents a healthy dimension of celebrity admiration.*”

*“**Therefore, the suitability of the CAS-7 on convenience samples, which are commonly used in celebrity worship research, needs further empirical evidence.”*

**3.Terms like "cross-cultural setting" and "applicability" are repeated multiple times. Consider varying word choice slightly for flow.**

Author’sresponse:

Thank you for pointing this out. We made some changes in the manuscrip taccordingly.

**Changes in themanuscipt:**

Page 8:

“*Therefore, the suitability of the CAS-7 on convenience samples, which are commonly used in celebrity worship research, needs further empirical evidence.*”

Page 9:

„*To date, no studies have provided evidence for the appropriateness of the CAS for cross cultural comparison purposes.”*

“*The aim of the current study was to test the psychometric properties of the CAS-7 in terms of factor structure and reliability in other cultures, thereby extending its potential use. To examine the consistency of the factor structure of the CAS-7, seven samples (student and fan convenience samples) from five countries (Canada, Hungary, Indonesia, Iran, and the United States) are used. This study also investigates the usability of the CAS-7 for cross-cultural research purposes if consistency across the factor structures is confirmed.*”

*“Evidence for the usability of the CAS-7 in a cross-cultural setting could contribute to a more accessible assessment of celebrity worship in future research and practice in a broader, international context.”*

Page 20:

*“However, to ensure the broader appropriateness and generalizability of previous findings regarding this brief measure, further validation across diverse cultural contexts is necessary.”*

*“Such evidence could provide further support for the international utility of the CAS-7 in different cultures.”*

Page 24:

“*Despite this limitation, preliminary evidence from five countries suggests that the CAS-7 has a consistent bifactor structure and good reliability, confirming its suitability for research purposes and practical utilization in a wider international context.”*

**4.While you hint at cultural differences (e.g., McCutcheon et al. finding differences between U.S. and India), you don't explain why cultural context might impact celebrity worship. Briefly explaining this would better justify the need for cross-cultural validation.**

Author’s response:

Thank you for this insightful observation regarding the need to elaborate on how cultural context may impact celebrity worship. We have now elaborated more on this aspect.

**Changes in the manuscript:**

Page 9:

“Cross-cultural studies using the CAS have been scarce. McCutcheon et al. (16) found substantial differences across students from different universities (i.e., U.S., India) in celebrity worship. *McCutcheon et al. (17)* *also found significant difference between African-American, Hispanic and Asian American, and White participants’ in celebrity worship levels. Specifically , African-American participants scored higher in the IP and BP dimensions than Hispanic and Asian American participants, while White participants had higher scores compared to African-American, Hispanic and Asian American participants. A strong preference was also demonstrated for celebrities of the same ethnicity to their own across these groups. McCutcheon et al. (17) explained that relating to a celebrity can offer a psychological escape from the difficult reality of belonging to a minority group in a society that cannot ascertain full acceptance toward these individuals.”*

**5.The transition to study aims feels a little abrupt. A bridge sentence summarizing why cross-cultural validation is needed based on previous limitations could strengthen it.**

Author’s response:

Thank you for pointing this out. We integrated a bridge sentence.

**Changes to the Manuscript:**

Page 9:

*„Building on previous findings, and the lack of a cross-cultural investigation of the CAS, the present study focuses specifically on the extension of the usability of the CAS across diverse populations.”*

**Introduction**

**1. How were the five countries (Canada, Hungary, Indonesia, Iran, and the U.S.) selected? Were there any cultural frameworks (e.g., individualism-collectivism) considered in their selection?**

Author’s response:

Thank you for your thoughtful question regarding the selection of countries. The five countries (Canada, Hungary, Indonesia, Iran, and the U.S.) were chosen based on the availability of relevant datasets and our established collaborations with research teams in these locations who are engaged in the study of celebrity worship and utilize the Celebrity Attitude Scale. The selection was therefore primarily based on convenience, reflecting accessible data sources and existing academic partnerships, rather than being guided by a specific cultural framework such as individualism-collectivism.

**Changes to the manuscript:**

Page 10:

„*The five countries (i.e., Canada, Hungary, Indonesia, Iran, and the USA) were selected based on existing research collaborations among research teams with significant expertise in celebrity worship research. Therefore, the selection was convenience-based.*”

**2. Since you're using student and fan samples, how comparable are these samples across countries? Are they matched on key demographics (age, gender)?**

Author’s response:

Thank you for raising this important point. To evaluate demographic comparability, we examined gender and age distributions across the seven samples. Chi-square and post hoc z-tests revealed significant differences in gender ratios with female proportions ranging from 27.1% (Iran) to 85.0% (Indonesia).

In terms of age, a one-way ANOVA showed significant differences across the samples in terms of age, *F*(6, 4346) = 245.74, *p*< .001. Tukey post hoc tests indicated that participants from Hungary (fan sample; *M* = 34.70) and the U.S. (general sample; *M* = 31.59) were significantly older than those in Indonesia (*M* = 19.45), U.S. students (*M* = 20.50), and Canada (*M* = 20.55).

These findings suggest that the samples had significant differences on key demographic variables. We now report these findings (see SM Table 7) acknowledge this in the revised manuscript and recommend that future studies either recruit more demographically balanced samples or include statistical controls for age and gender when examining cross-cultural effects.

**Changes to the manuscript:**

Page 10:

*„A chi-square test revealed a significant difference in gender distribution across samples, χ²(12, n = 4353) = 1035.04, p < 0.001, with the Iranian sample having the highest proportion of male participants (72.9%) and the Indonesian sample having the highest proportion of females (85.0%). Similarly, a one-way ANOVA showed significant differences in participants’ age across groups, F(6, 4346) = 245.74, p < 0.001. The youngest mean age was demonstrated in the Indonesian student sample (M_age_ = 19.45, SD = 1.89), while the highest mean age was found in the Hungarian fan sample (M_age_ = 34.70, SD = 12.40) (see SM Table 7).”*

Page 23:

*“**Another important limitation of this study relates to the heterogeneity of the samples. Demographic variability may further limits the generalizability of the present findings.”*

**3. Was measurement invariance testing (e.g., configural, metric, scalar invariance) considered to formally establish cross-cultural comparability of the CAS-7?**

Author’s response:

Thank you for this valuable comment. To assess cross-cultural comparability of the CAS-7, we conducted measurement invariance testing using a stepwise approach (configural, metric, scalar) across six country samples where the two-factor model showed acceptable fit. While the configural model demonstrated good fit (*χ*²(78) = 224.26, *p* < 0.001; CFI = .981; RMSEA = .055), subsequent models indicated declining fit indices. The metric model showed moderate change (ΔCFI = .018; ΔRMSEA = .011), and the scalar model yielded a notable decline in fit (ΔCFI = .024; ΔRMSEA = .051), suggesting that full scalar invariance was not supported. Given these findings and the known limitations of bifactor models in cross-cultural comparisons due to differential constraints, we opted to retain the more stable and theoretically grounded two-factor structure for the current study. These limitations and theoretical implications are now discussed in the manuscript.

**Changes to the manuscript:**

Page 13-14:

*“To examine the cross-cultural comparability of the CAS-7, stepwise measurement invariance testing was employed using multigroup CFA (24) across six country samples in which the two-factor model showed acceptable fit. The Iranian sample was excluded from this analysis as the two-factor model did not show good fit in this sample. The grouping variable was the origin of the sample (i.e., country). Although the bifactor model showed superior fit compared to the two-factor model across some of the samples, due to the lack of differences in the measurement model (i.e., different item-level restrictions) which were present in the bifactor models, alongside with theoretical interpretability (see 14), stability and replicability across the present samples, and good psychometric properties of the two-factor model, this model was selected for the invariance testing. The two-factor model was comparable across the samples as the measurement model was equal. Configural, metric, and scalar invariance were sequentially tested to detect potential measurement biases. Nonsignificant change in the commonly used fit indices indicates measurement invariance in the indicated levels (25,26): ΔCFI ≤ 0.010; ΔTLI ≤ 0.010; and ΔRMSEA ≤ 0.015; ΔSRMR ≤ 0.030 for metric and 0.015 for scalar invariance.”*

Page 18:

*“Measurement invariance testing based on the two-factor model across the six country samples did not support either metric or scalar invariance (see Table 4). Specifically, changes in the fit indices (i.e., CFI, TLI, RMSEA, SRMR) were substantial across the scalar–metric, and metric–configural models. Therefore, measurement invariance was not demonstrated across the samples. In more detail, the factor loadings and the intercepts (mean scores) are substantially varied across the samples, preventing meaningful cross-cultural comparison in celebrity worship levels measured by the CAS-7. In summary, the CAS-7 measure does not meet the criteria for cross-cultural comparability in terms of mean scores and strength of associations between celebrity worship and other constructs, despite the consistent and replicable factor structures across the cultural samples.”*

Page 21:

“*In summary, the present findings suggest that the CAS-7 is a psychometrically sound tool for assessing celebrity worship separately in diverse cultural contexts, as the bifactor structure demonstrated good overall fit across each sample.* *Measurement invariance testing did not support metric and scalar invariance, suggesting that neither factor loadings, nor intercepts were equivalent across the samples, seriously limiting the scale’s appropriateness for cross-cultural comparisons in terms of associations or latent means.”*

Page 23:

*“Finally, neither metric, nor scalar invariance was supported across the examined country samples. Items and item groups contributing to the lack of measurement invariance were investigated by analyzing the invariance of the two factors of the CAS-7 separately. Evidence of metric or scalar invariance was not found for either factor. Therefore, we were unable to identify any item group that could be invariant across the examined samples. Future research may consider applying further item-level analysis (e.g., item response theory methods).”*

**4. If CAS-7 is found to be "fundamentally unidimensional," how should future researchers treat the subscales — still interpret separately or focus mainly on the total score?**

Author’s response:

Thank you for raising this important methodological consideration. While the CAS-7 demonstrates fundamental unidimensionality, we recommend that researchers interpret both subscales (*entertainment-social* and *intense-pathological*) alongside the total score. These subscales capture distinct dimensions of celebrity worship (e.g., social bonding vs. excessive preoccupation), which can remain meaningful despite the scale’s unidimensional structure, based on the respective research aims. The choice to prioritize subscales or the global score should depend on the study’s focus: subscales are preferable for examining mechanisms linked to specific behaviors (e.g., differentiating healthy engagement from problematic levels of admiration), while the total score is suitable for assessing general celebrity admiration. This approach aligns with recent psychometric recommendations advocating for context-driven interpretations of unidimensional measures (see Zsila et al., 2024). We have clarified this rationale in the revised manuscript.

**Changes to the manuscript:**

Page 20:

*"Researchers should use the subscale or the total score of the CAS-7 based on the study’s objectives. Specifically, subscales could be used to provide a more differentiated picture on the associations of specific behavioral or emotional dimensions (e.g., excessive absorption or healthy interest in a celebrity) with other psychological constructs (e.g., cognitive functioning), and the global score for assessing celebrity admiration when the research aim is rather focused on measuring the extent of celebrity admiration generally (see (14)).*

**5. If CAS-7 shows poor fit in certain countries, would you recommend culturally adapting it, or would you suggest developing entirely new culturally sensitive scales?**

Author’s response:

Thank you for raising this critical methodological question. Our analyses revealed no instances of poor model fit for the CAS-7 across the five countries studied. While the bifactor model demonstrated superior fit in all cultural contexts (including Iran, where alternative structural configurations showed marginally better but statistically equivalent performance), we emphasize that the scale’s core unidimensional structure remained stable. Therefore, it is not necessary to develop other scales because of cultural differences, as the present measure works properly in the investigated cultural samples, only cross-cultural comparisons are limited.

**Changes to the manuscript:**

Page 24:

*„**Given that the bifactor model demonstrated consistently good fit across all samples, this model structure can be used in various cultural contexts without the need for any culture-specific modifications. However, the lack of both metric and scalar invariance indicates that future studies may benefit from investigating the cross-cultural application of the CAS-7 further by analyzing its psychometric properties and testing invariance in other cultural contexts (e.g., more European and Asian countries) to broaden cultural usability perspectives.”*

**Methods**

**1. Convenience sampling inherently limits generalizability, but this limitation is not acknowledged. It should be.**

Author’s response:

Thank you for your careful reading and valuable feedback. We agree that the use of convenience sampling limits the generalizability of our findings. We have now made this limitation even more explicit in the revised manuscript.

**Changes to the manuscript:**

Page 22-23:

*"The use of convenience sampling restricts the generalizability of the results, as the sample may not be representative of the broader populations in the participating countries."*

**2. How many participants came from each country/sample (students vs general population vs celebrity fans)?**

Author’s response:

Thank you for your question. The number of participants from each country and sample group (students, general population, fans) who fully completed the questionnaire is detailed in Table 1 of the manuscript. Only full completions were administered, so no further exclusion criteria (e.g., not full completion) could be reported.

**3. Was the sample distribution approximately equal across groups, or heavily skewed toward one?**

Author’s response:

Thank you for your question. The sample distribution was not equal across countries or subgroups, and we fully acknowledge this as a limitation. As detailed in the revised manuscript (page 16, Limitations section), significant demographic disparities existed (see Comment 2 for Introduction):

- Sample size imbalance: Participant numbers varied considerably across countries (see Table 1).
- Gender distributions: Female representation ranged from 27.1% (Iran) to 85.0% (Indonesia), with significant differences in the sample comparisons.
- Age differences: Hungarian fan (M = 34.70) and U.S. general (M = 31.59) samples were substantially older than Indonesian (M = 19.45) and student samples (Tukey tests, *p* < .001).

**4. It is unclear why data access dates are different from data collection dates.
o Line 182–183: "The data for research purposes were accessed between 31/12/2015 and 12/04/2024." But participants were recruited starting from 2018. Why access data from 2015?
o Please clarify whether there were existing datasets before new data collection started. •**

Author’s response:

We apologize for the mistake in reporting the data collection and access dates. We have clarified this in the revised manuscript.

**Changes to the manuscript:**

Page 11:

*„The data analyzed in this study were collected between 26/11/2018 and 02/03/2024. No information was available that could personally identify participants. Data for the secondary analysis were available between 01/01/2024 and 12/04/2024.”*

**5. Other than age and gender, were any other sociodemographic variables collected (e.g., education, income, country of residence)? If yes, mention them.**

Author’s response:

Thank you for your question. In addition to age and gender, we collected information on participants’ country of residence. No other sociodemographic variables, such as education or income, were assessed in this study.

**6. Why is this large sample appropriate for CFA and SEM? (A brief note would strengthen the methods section.)**

Author’s response:

Thank you for highlighting this methodological consideration. While sample size guidelines for SEM vary, our study’s large samples exceed established thresholds for robust analyses. For instance, Comrey and Lee (1992) classify samples above 300 as "good" for factor analysis, and recent SEM studies report median sample sizes of ~200 (Kline, 2016). Our sample size ensures sufficient statistical power, minimizes bias in parameter estimates, and enhances the stability of the model structure. We have added a concise justification to the Methods section referencing these guidelines.

**Changes to manuscript:**

Page 13:

**„***As each country sample included more than 200 participants, group sizes exceeded established recommendations for factor analysis and SEM, ensuring sufficient statistical power and stable parameter estimates (19,20).*”

**7. Why are the data access dates (starting from 2015) earlier than the participant recruitment dates (2018)? Were earlier datasets used? Please clarify.**

Author’s response:

Thank you for this important clarification request. For the current study, no datasets prior to 2018 were accessed or used. The actual participant recruitment for the data used in this analysis occurred between 2018 and 2024. To avoid confusion, we have revised the manuscript to indicate that only data collected and accessed between 2018 and 2024 were included in the current analysis. The changes made tot he manusript can be seen on page 11 (see the earlier comment for Question 4).

**8. Since participants were from five different countries, was the CAS-7 translated into local languages?**

Author’s response:

Thank you for your question regarding the translation of the CAS-7. Yes, the CAS-7 was translated into the relevant local languages-English, Hungarian and Persian, -following established cross-cultural translation procedures for self-report measures. We have added this information to the Methods section as well.

**Changes to the manuscript:**

Page 12:

„*For all versions that were not originally administered in English, the questionnaires were translated and back-translated to English ensure conceptual and linguistic equivalence*. *Specifically, Hungarian translation was available for Hungarian participants, and Persian version was administered for Iranian participants.*”

**9. Could you please define ES and IPBP when first introducing them? It will help readers unfamiliar with the original CAS structure.**

Author’s response:

Thank you for your suggestion. We have now clarified in the Results section that ES refers to the Entertainment–Social dimension, and IPBP refers to the combined Intense–Personal and Borderline–Pathological dimension of the CAS-7.

**Changes to the manuscript:**

Page 14:

“*The 2-factor models with two intercorrelated celebrity worship factors (i.e., ES (Entertainment-Social) and IPBP (Intense-Pathological) - comprised items from the IP (Intense-Personal) and BP (Borderline-Pathological) theoretical factors) showed an excellent fit with the exception of the Iranian sample (n = 627).”*

**Discussion**

**1. Ideas sometimes jump between results, limitations, and broader interpretations without clear transitions. Use more structured subsections or clearer paragraph transitions.**

Author’s response:

Thank you for your constructive feedback. In response, we have revised the Discussion section by organizing it into clearly structured subsections to improve flow and readability.

**2. The claim that CAS-7 “may be widely applicable” (lines 310–311) feels overstated, especially given the cross-cultural limitations admitted. Temper this language to avoid implying broader validity than supported.**

Author’s response:

Thank you for raising your concerns – we made the necessary clarification in the manuscript.

**Changes to the manuscript:**

Page 23:

*„Overall, the current study provides some preliminary evidence, supporting the potential applicability of the CAS-7 in diverse cultural contexts separately.”*

Page 24:

*„However, the lack of both metric and scalar invariance indicates that future studies may benefit from investigating the cross-cultural application of the CAS-7 further by analyzing its psychometric properties and testing invariance in other cultural contexts (e.g., more European and Asian countries) to broaden cultural usability perspectives.”*

**3. The Iranian sample's deviation is mentioned but not discussed in depth. Elaborate more on why the Iranian sample's results differed—was it sample size, cultural interpretation of celebrity, translation issues, etc.?**

Author’s response:

Thank you for this critical observation. While the bifactor model demonstrated good fit in the Iranian sample, the marginally better performance of an alternative structural configuration likely stems from methodological and contextual factors rather than fundamental cultural differences:

- **Prior Validation**: A recent Persian CAS validation (Ahadi et al., 2025) also identified unidimensionality via EFA but did not test bifactor models. Our findings extend this work by demonstrating bifactor viability.
- **Sample Size**: The Iranian subsample (n = 627) was smaller than some of the other groups, potentially reducing statistical power to detect subtle structural nuances.
- **Cultural Context:** Iran’s media landscape—with strict regulations on celebrity representation—may shape distinct fandom expressions (e.g., stronger emphasis on religious or political figures). However, the CAS-7’s core structure remained stable, suggesting cross-cultural validity.

**Changes to the manuscript:**

Page 21:

*“**The two-factor model did not show good fit to the data in the Iranian sample, which may be attributed to methodological issues (e.g., small sample size) rather than fundamental cultural differences, as evidenced by the bifactor model’s robust fit.”*

**4. Besides research, could CAS-7 be used for clinical, media, or public health purposes in different countries? Briefly discuss practical applications and caution about their limitations due to cultural variability.**

Author’s response:

Thank you for this insightful question about the CAS-7’s practical utility. While primarily a research tool, the CAS-7 may have potential applications in:

- **Clinical Settings**: Screening and identification of excessive celebrity worship linked to subclinical distress (e.g., anxiety, compulsive behaviors) or comorbid conditions (e.g., mood disorders). However, it should not be used as a standalone diagnostic tool.
- **Media/Entertainment**: Identifying fan engagement patterns to tailor content or marketing strategies.
- **Public Health**: Informing campaigns about healthy vs. problematic fandom behaviors, particularly targeting young adults.

These applications may require careful cultural considerations, as norms around celebrity engagement vary significantly (e.g., collectivistvs. individualist societies). For instance, CAS-7 scores indicating "pathological" worship in one culture might reflect normative social bonding in another.

**Changes to the manuscript:**

Page 21:

*"**While its applicability for cross-cultural comparisons was not supported in the present study, the CAS-7 may nonetheless prove to be a valuable tool in psychological practice and research for identifying individuals with elevated celebrity worship tendencies and exploring their mental health correlates. Besides its serious limitations regarding cultural non-invariance, the scale’s use in different cultural contexts also requires careful interpretation, as cultural norms may shape both fandom expressions and thresholds for considering a fan engagement as problematic.”*

**5. It’s unclear how different participant sources (students, general public, celebrity fans) might have influenced the findings. Reflect briefly on whether sample type (beyond nationality) may have introduced additional variability.**

Author’s response:

Thank you for raising this critical point. The inclusion of diverse sample types (students, general population, fans) might introduced additional variability, as these groups differ in their engagement with the celebrity culture. For instance, fan samples may exhibit higher baseline CAS-7 scores due to their possibly high engagement, while student samples might rather reflect age-specific media consumption patterns. We added a sentence to the manuscript, to clarify this limitation as well.

**Changes to the manuscript:**

Page 23:

*„Additional variability may have arisen from the inclusion of fan, general population, and student samples, which may differ not only in demographic characteristics but also in the level of engagement with the celebrity culture and patterns of popular media content consumption.”*

**6. What are your hypotheses for why the two-factor model did not fit well for the Iranian sample? Could it be related to cultural norms about fame or admiration?**

Author’s response:

Thank you for revisiting this important issue. As discussed in the manuscript (see Page 16), the two-factor model did not fit the Iranian sample as well as in other groups. However, this variation appears to reflect methodological and contextual factors—such as sample characteristics and local media regulation—rather than fundamental cultural incompatibility. Notably, the bifactor model showed robust fit in the Iranian sample, suggesting that the CAS-7's core structure remains applicable across cultural contexts, even where norms surrounding celebrity and fame differ.

**7. Were there particular CAS-7 items that performed inconsistently across cultures? If yes, which ones and why might that be?**

Author’s response:

Thank you for this insightful question. While full scalar invariance was not achieved, we did not conduct further item-level analyses to identify which specific items might have contributed to this result. A more granular exploration, such as using item-response theory (IRT), would have substantially increased the scope and length of the present manuscript. We acknowledge this as a limitation and suggest that future studies may explore item-level functioning across cultures in greater depth.

**Changes to the manuscript:**

Page 23:

*“**Finally, neither metric, nor scalar invariance was supported across the examined country samples. Items and item groups contributing to the lack of measurement invariance were investigated by analyzing the invariance of the two factors of the CAS-7 separately. Evidence of metric or scalar invariance was not found for either factor. Therefore, we were unable to identify any item group that could be invariant across the examined samples. Future research may consider applying further item-level analysis (e.g., item response theory methods).”*

**Reviewer**

**1. Dear authors, I found your work invaluable and a timely issue however I have a suggestion for you. Despite mentioned in problem statement slightly, you did not justify 7-point likert scale than a 5-point likert scale. I believe that you need to critically show the rationale of using CAS-7 factor structure with its implications under the method section- measure section.**

Author’s response:

Thank you for your careful reading and valuable feedback. We would like to clarify that the CAS-7 was administered using **a 5-point Likert scale** (1 = *strongly disagree* to 5 = *strongly agree*), consistent with prior validations of the CAS scale (McCutcheon et al., 2002; Zsila et al., 2024). No deviations from the original response format were implemented in this study. We have revised the Methods section and found no information regarding a 7-point scale.
